# Differences in patient population and service provision between nurse practitioner and general practitioner consultations in Swiss primary care: a case study

**DOI:** 10.1186/s12875-020-01240-8

**Published:** 2020-08-13

**Authors:** Stefan Gysin, Rahel Meier, Anneke van Vught, Christoph Merlo, Armin Gemperli, Stefan Essig

**Affiliations:** 1 Institute of Primary and Community Care, Schwanenplatz 7, 6004 Luzern, Switzerland; 2 Department of Health Sciences and Medicine, University of Lucerne, Luzern, Switzerland; 3 Institute of Primary Care Zurich, University of Zurich and University Hospital Zurich, Zürich, Switzerland; 4 HAN University of Applied Sciences, Faculty of Health and Social Studies, Nijmegen, the Netherlands; 5 Swiss Paraplegic Research, Nottwil, Switzerland

**Keywords:** Primary care, Nurse practitioner, Advanced practice nurse, Advanced nursing practice, General practitioner, Family practice, Multimorbidity, Polypharmacy, Elderly

## Abstract

**Background:**

Primary care systems around the world have implemented nurse practitioners (NPs) to ensure access to high quality care in times of general practitioner (GP) shortages and changing health care needs of a multimorbid, ageing population. In Switzerland, NPs are currently being introduced, and their exact role is yet to be determined. The aim of this study was to get insight into patient characteristics and services provided in NP consultations compared to GP consultations in Swiss primary care.

**Methods:**

This case study used retrospective observational data from electronic medical records of a family practice with one NP and two GPs. Data on patient-provider encounters were collected between August 2017 and December 2018. We used logistic regression to assess associations between the assignment of the patients to the NP or GP and patient characteristics and delivered services respectively.

**Results:**

Data from 5210 patients participating in 27,811 consultations were analyzed. The average patient age was 44.3 years (SD 22.6), 47.1% of the patients were female and 19.4% multimorbid. 1613 (5.8%) consultations were with the NP, and 26,198 (94.2%) with the two GPs. Patients in NP consultations were more often aged 85+ (OR 3.43; 95%-CI 2.70–4.36), multimorbid (OR 1.37; 95%-CI 1.24–1.51; *p* < 0.001) and polypharmaceutical (OR 1.28; 95%-CI 1.15–1.42; p < 0.001) in comparison to GP consultations. In NP consultations, vital signs (OR 3.05; 95%-CI 2.72–3.42; *p* < 0.001) and anthropometric data (OR 1.33; 95%-CI 1.09–1.63; p 0.005) were measured more frequently, and lab tests (OR 1.16; 95%-CI 1.04–1.30; p 0.008) were ordered more often compared to GP consultations, independent of patient characteristics. By contrast, medications (OR 0.35; 95%-CI 0.30–0.41; p < 0.001) were prescribed or changed less frequently in NP consultations.

**Conclusions:**

Quantitative data from pilot projects provide valuable insights into NP tasks and activities in Swiss primary care. Our results provide first indications that NPs might have a focus on and could offer care to the growing number of multimorbid, polypharmaceutical elderly in Swiss primary care.

## Background

Primary care systems around the world have introduced nurse practitioners (NPs) with the goal to ensure access to high quality care in times of provider shortages, increasing demands and changing health care needs of a multimorbid, ageing population [1]. Depending on the country, NPs hold a master’s or doctoral degree and have acquired the expert knowledge base, complex decision-making skills and clinical competencies for expanded practice [2]. The NP role originated in the US in the 1960s to improve access to primary health care in rural areas [3]. In the Netherlands, the main driver to introduce NPs to primary care in the 1990s was the shortage of general practitioners (GPs). NPs were supposed to provide substitution and supplementation for minor ailments in order to reduce the GPs’ caseload [4]. In other countries, such as Singapore, the NP role was implemented to increase the attractiveness of the nursing profession, and to retain experienced nurses in clinical practice [5]. Depending on the legislation of the country, the NP’s scope of practice includes clinical assessment, ordering and interpreting of diagnostic tests, diagnosis-making, providing treatment including prescribing as well as educating and counseling patients autonomously [6]. NPs provide primary, acute, chronic and specialty care to patients of all ages and in all health care settings [7]. In some countries like the US, Canada and the UK, they can also specialize on specific patient groups such as children or women, and there are nurse-led clinics and general practices [1]. Maier et al. [8] found that NPs are able to provide up to 90% of all primary care services, and therefore show high potential to substitute physician tasks. Overall, studies from the US, Canada, the UK and the Netherlands show that NPs in primary care achieve slightly higher patient satisfaction, patient enablement, and provide equal or possibly better quality of care for first contact, ongoing physical complaints as well as follow-up care for specific chronic conditions such as diabetes compared to GPs [9]. NPs also seem to have longer consultations and make more investigations than doctors [10]. In addition, Barratt et al. [11] found that NPs use a more personable, everyday style of communication, which can optimize interactions with patients and improve shared clinical decision-making.

Switzerland is in the early stages of introducing the NP role. The first Master of Science in Nursing program was implemented in 2000, and initially focused on research and organizational leadership [12]. Since 2015, educational programs at master’s level as well as postgraduate programs have focused more on clinical skills and competencies with the goal to better prepare the students for the NP role. Despite the political interest for new care models in lights of expected GPs shortages in rural areas, there are currently only a handful of ongoing NP pilot projects in primary care with the goal to address the health care needs of the increasing numbers of multimorbid elderly [13, 14]. One reason for the low number of projects might be that there is no legal framework which defines the NPs’ scope of practice, accountability or reimbursement options [12]. Under consideration of the current federal law, NPs are allowed to adjust and prescribe certain medications under the delegation of physicians, according to a legal opinion from 2016 [15]. Nationally, self-organized professional working groups are aiming at putting further regulations in place to spark discussions and to promote the NP role [16]. Furthermore, Bryant-Lukosius and colleagues [17] proposed a framework for evaluating the impact of advanced practice nursing roles in Switzerland. They suggested to identify characteristics of patients to be the focus of the NP role, and to assess health care services delivered by NPs in the early stages of role introduction. So far, evidence about NPs’ impact on Swiss primary care is mostly hypothetical [18], published in non-peer reviewed journals [19] or of qualitative nature [14, 20]. First results from these studies indicate that NPs might have a focus on older patients with chronic conditions, and may deliver high quality care. However, quantitative studies are lacking, and most stakeholders, including GPs, still only have a vague idea about the potential NP role.

The aim of this study was to gain insight into patient characteristics and services delivered in NP consultations compared to GP consultations in Swiss primary care.

## Methods

### Study design

This is a case study using retrospective observational data from electronic medical records (EMRs) of a family practice in Swiss primary care. We followed the definition of a case study as a detailed and in-depth analysis of a particular case or several cases considering the contextual factors of the unit of analysis, which can be one or many individuals or an organization such as a practice [21].

### Case setting, data collection and study population

In this family practice, run by two experienced GPs, the local health authority initiated a pilot project with the goal to counteract GP shortages and to ensure access to care for potentially underserved patients in the rural area using a new interprofessional model of care. The project started in August 2017 with the employment of a part-time working NP who was simultaneously completing a postgraduate continuous education to improve her clinical skills and competencies, especially in the complex care of elderly [22]. The NP had worked as a registered nurse for about 20 years before obtaining her master’s degree in 2011. After graduation, she worked in the hospital and mainly provided leadership, care coordination and quality control. The NP had no previous experience in primary care before the start of the project. The two GPs who provided clinical supervision were not familiar with the NP model beforehand but received specific information regarding the NP role and education by the project team.

The practice participated in the FIRE (Family medicine ICPC Research using EMR) project, a research database based on a network of Swiss GPs contributing anonymized medical routine data on patient-provider encounters extracted from electronic medical records (EMRs) [23]. We collected and analyzed data from the FIRE database over a period of 17 months between August 2017 and December 2018. The NP coded her consultations, which allowed us to identify all her patient encounters and separate them from those of the two GPs. Patients who received at least one consultation at the practice during the data collection period were included.

### Variables and measures

For each consultation, we retrieved information on patient characteristics (age, sex, multimorbidity status and number of different drugs taken) and services delivered (measurement of vital signs, i.e. pulse and/or blood pressure; measurement of anthropometric data, i.e. weight, height and/or BMI; ordering of lab tests, e.g. HbA1c; prescription and/or change of medication). Age was divided into six categories. Multimorbidity status was determined at baseline and defined as the presence of two or more chronic conditions over the whole data collection period. Chronic conditions were determined based on prescribed drugs, vital signs, anthropometric data and/or lab values (see additional file 1). Polypharmacy was determined in each consultation and defined as more than four active medications.

### Data analysis

Baseline characteristics of the study population are presented as means for continuous variables, and as percentages for categorical variables. To assess associations with the assignment of the patient to the NP or GP, we used a logistic regression model. The consultation assignment was treated as a dependent variable, and patient characteristics and delivered services as independent variables. For each variable in the model, we adjusted for age, sex, multimorbidity and polypharmacy. The results are presented as unadjusted and adjusted odds ratios (OR) with 95% confidence intervals (95% CI) and *p* values. The alpha level for statistical significance was set at < 0.05. To calculate the global p value for the age categories, we performed a likelihood ratio test. Statistical analyses were carried out using Stata 15.0.

## Results

### Study population

In total, 5210 patients were eligible to be included in the study, i.e. had at least one consultation with the NP and/or the two GPs in the data collection period. The average patient age was 44.3 years (SD 22.6), 47.1% of the patients were female and 19.4% multimorbid. Over the 17 months data collection period, patients had an average of 5.3 (SD 6.6) consultations at the family practice. 969 (18.6%) patients had at least one consultation with the NP (mean = 1.7; SD 2.1) and 5129 (98.4%) patients had at least one consultation with one of the two GPs (mean = 5.1; SD 6.2). Altogether, we analyzed data from 27,811 consultations. 1613 (5.8%) of these consultations were with the NP, and 26,198 (94.2%) with the two GPs. Table 1 gives a detailed overview of the consultations.

**Table 1:** Comparison of nurse practitioner (NP) and general practitioner (GP) consultations

	NP consultations *N* = 1613 n (column %)	GP consultations *N* = 26,198 n (column %)	Crude odds ratio (95% CI)	P value crude odds ratio	Adjusted odds ratio* (95% CI)	P value adjusted odds ratio
***Consultations with***						
Patients aged				< 0.001**		< 0.001**
0–17 years	92 (5.7)	2412 (9.2)	1		1	
18–34 years	253 (15.7)	4518 (17.3)	1.47 (1.15–1.87)		1.48 (1.16–1.88)	
35–54 years	333 (20.6)	6473 (24.7)	1.35 (1.07–1.71)		1.34 (1.06–1.70)	
55–64 years	229 (14.2)	3837 (14.7)	1.56 (1.22–2.00)		1.53 (1.18–1.97)	
65–84 years	394 (24.4)	6573 (25.1)	1.57 (1.25–1.98)		1.55 (1.21–2.00)	
85+ years	312 (19.3)	2385 (9.1)	3.43 (2.70–4.36)		3.48 (2.66–4.55)	
Male patients	829 (51.4)	13,267 (50.6)	1.03 (0.93–1.14)	0.557	1.09 (0.99–1.21)	0.075
Multimorbid patients	816 (50.6)	11,212 (42.8)	1.37 (1.24–1.51)	< 0.001	1.11 (0.96–1.28)	0.148
Polypharmaceutical patients	569 (35.3)	7824 (29.9)	1.28 (1.15–1.42)	< 0.001	0.89 (0.77–1.03)	0.114
***Consultations included***						
Blood pressure / pulse measurement(s)	518 (32.1)	3495 (13.3)	3.07 (2.75–3.43)	< 0.001	3.05 (2.72–3.42)	< 0.001
Weight / height / BMI measurement(s)	112 (6.9)	1486 (5.7)	1.24 (1.02–1.51)	0.034	1.33 (1.09–1.63)	0.005
Laboratory testing	490 (30.4)	7325 (28.0)	1.12 (1.01–1.25)	0.036	1.16 (1.04–1.30)	0.008
Prescription / change of medications	210 (13.0)	7724 (29.5)	0.36 (0.31–0.41)	< 0.001	0.35 (0.30–0.41)	< 0.001

* adjusted for age, sex, multimorbidity and polypharmacy; ** global *p*-value (calculated using likelihood ratio test); “/” = and/or

### Associations of patient characteristics and NP consultations

In their consultations, both the NP and GPs saw patients of both sexes and from all age groups. However, NP consultations were associated with higher patient age compared to the GPs’ consultations (global *p* < 0.001). This was particularly distinct in the group of patients older than 85 years (OR 3.43; 95%-CI 2.70–4.36). NP consultations were also associated with a significantly higher share of multimorbid (OR 1.37; 95%-CI 1.24–1.51; *p* < 0.001) and polypharmaceutical (OR 1.28; 95%-CI 1.15–1.42; p < 0.001) patients. After adjusting for the potential confounders age, multimorbidity and polypharmacy, age was still significantly associated with NP consultations (adjusted p < 0.001) but multimorbidity (adjusted OR 1.11; 95%-CI 0.96–1.28; p 0.148) and polypharmacy (adjusted OR 0.89; 95%-CI 0.77–1.03; p 0.114) were not.

### Associations of delivered services and NP consultations

The NP and GPs offered all four services (i.e. measurement of vital signs and anthropometrics, ordering of lab tests and prescription/change of medications) in their consultations. There were significant associations between NP consultations and service provision. In comparison to GP consultations, vital signs (OR 3.07; 95%-CI 2.75–3.43; *p* < 0.001) and anthropometric data (OR 1.24; 95%-CI 1.02–1.51; p 0.034) were measured more frequently, and lab tests were ordered more often (OR 1.12; 95%-CI 1.01–1.25; p 0.036) during NP consultations. On the other hand, medications were prescribed or changed less often in NP consultations compared to GP consultations (OR 0.36; 95%-CI 0.31–0.41; p < 0.001). After adjusting for the potentially confounding factors age, multimorbidity and polypharmacy, all four services remained significantly associated with NP consultations (Table 1).

## Discussion

### Main findings

Overall, NP consultations were associated with higher patient age, and a higher share of multimorbid and polypharmaceutical patients in comparison to GP consultations. Age remained significantly associated with NP consultations after adjusting for potential confounders, multimorbidity and polypharmacy did not. During NP consultations, vital signs and anthropometric data were measured more frequently, and lab tests were ordered more often. By contrast, medications were prescribed or changed more frequently in GP consultations than in NP consultations.

### Interpretation & comparison to existing literature

Bryant-Lukosius et al. [17] suggested in their evaluation framework “PEPPA Plus” to determine the characteristics of patients seen and treated by Swiss NPs in the early stages of role introduction. Our results indicate that the NP had a focus on multimorbid, polypharmaceutical elderly. In fact, almost 20% of her consultations were with patients aged 85+. This might be a consequence from the NP’s postgraduate education, which focused on care for older patients with complex health care needs [22]. Another reason could be that these patients were potentially underserved in this practice, and might have been specifically assigned to her within the project. In a qualitative study by Gysin et al. [14], most NPs who work in Swiss family practices reported a similar patient population they served. This goes in line with current political efforts to address the increase in chronic conditions. International studies showed that NPs provide at least equivalent care for people with chronic conditions as physicians, often through patient education, multidimensional assessments and coordination of multiple providers [24, 25]. In some countries such as Sweden, NPs also have a focus on chronically ill elderly [26]. In other countries like the US, Canada, Australia and the UK, NPs treat patients across the age span and take care of minor acute illnesses as well as chronic conditions [10, 27–29]. For instance, in Veterans Health Administration facilities, Morgan et al. [30] found that patient age did not differ between NP and GP consultations in primary care offices. However, in the US, NPs can specialize in gerontology, and a study by Hendrix et al. [31] found that these geriatric NPs might be the most appropriate providers of coordinated chronic care to the elderly population. Interestingly, a Dutch study from Van Der Biezen et al. [32] showed that GPs saw more patients aged 65+ in comparison to the NPs. However, this study analyzed out-of-hours primary care consultations. Therefore, comparability might be limited.

Multimorbid, polypharmaceutical elderly are often homebound, and it is possible that the NP in our study conducted more home visits, including visits in nursing homes, than the GPs, which could explain the higher patient age in her consultations. In the US, NPs are the largest type of “full time house call providers” with prescriptive authority [33]. In Switzerland, the number of GP home visits have decreased drastically in recent years, and home visits, especially follow-up visits to elderly, have been identified as a task that could potentially be performed by NPs [34]. The NP in our study measured vital signs and anthropometric data or assigned these tasks to practice assistants more frequently compared to the GPs, and ordered lab tests more often. This might be because the NP saw more multimorbid, polypharmaceutical elderly, which usually need closer monitoring, e.g. regular blood pressure measurements in hypertension, weight control in heart failure or frequent HbA1c measurements in diabetes. However, the significant differences remained after adjusting for age, multimorbidity and polypharmacy. This could have several reasons. As a novice and pioneer, the NP was maybe more careful and ordered clinical and lab parameters more often in order not to miss something. Several pioneering NPs in Swiss primary care mentioned similar behavior before [14]. International studies found that nurse-led care can result in improved blood pressure control and outcomes, e.g. in diabetes care or cardiovascular prevention [35, 36]. These findings were often attributed to stricter guideline adherence. Similarly, Chan et al. [37] found that NP care for patients who suffered from dyspepsia and underwent gastroscopy was effective because of the adherence to standardized follow-ups which included weight measurement. Ohman et al. [38] found that practices with NPs were more likely to measure lab values (e.g. HbA1c, lipid levels or urinary microalbumin levels) compared to practices with physicians and physician assistants or physicians only. These findings are in accordance with our study results.

The two GPs in our study changed and prescribed new drugs more often than the NP. This could be explained by the fact that NPs do not have independent, full prescription rights in Switzerland yet, and educational programs still lack several hours on pharmacology compared to international standards, which could yield in hesitation of prescribing new drugs. De Bruijn-Geraets et al. [39] found that prescription rates of Dutch NPs increased after obtaining full legal practice authority. However, during out-of-hours consultations, Van Der Biezen et al. [32] found that NPs still prescribed less medications compared to GPs. The authors hypothesized that this could result from more patient education. In the UK, nurses can obtain independent prescribing rights after undergoing the necessary training, and several studies have been conducted regarding health outcomes of “non-medical” prescribing [40–42]. Venning et al. [43] found no difference between prescription rates of NPs and GPs in the UK. This aligns with the findings of an international systematic review by Laurant et al. [9], which is mostly based on studies from countries at advanced stage of NP role implementation. Furthermore, in the US, Barnes et al. [44] found that independent prescription rights for NPs (i.e. same rights as doctors) may lead to higher participation of NPs in primary care. This finding could be relevant when implementing the NP role in Swiss primary care.

### Limitations

We only had data from one family practice with one NP, which limits the external validity of this study as it was influenced by personal factors (e.g. the NP’s previous experience as a registered nurse) and politically-driven project factors (e.g. the goal to address chronically ill elderly). However, these political factors might reflect what is considered important when new professionals are introduced to a health care system, and may be present even if larger cohorts are investigated. We measured patient characteristics and activities during NP consultations but did not assess or compare the quality of care itself. Further, the practice did not use ICPC-2 codes; hence, we did not have any information on the reasons for encounter. However, Busato et al. [45] showed that using drugs to identify morbidity within FIRE data is as reliable as using ICPC-2 codes. We did not know how much the NP’s activities were influenced by the two supervising GPs, and we could not assess to what extent the NP complemented or substituted the GPs. We could also not measure whether certain activities (e.g. blood pressure measurement) were actually performed by the NP and GPs respectively, or by a practice assistant. There were not sufficient information regarding the site of the consultations; hence, we did not know which consultations took place in the practice or at a patient’s home. Lastly, we could not assess whether missing information was due to non-performance or non-documentation in the EMR. This limitation has been discussed by Djalali et al. [46] when using FIRE data.

## Conclusions

Quantitative data from pilot projects provide valuable insights into the NP tasks and activities in Swiss primary care. These insights might trigger suitable regulations and promote further role implementation. Standardized curricula with more pharmacology, and defined scope of practices could allow NPs to focus on a certain groups of patients and prescribe certain drugs more independently, i.e. without GP supervision. This could then lead to more role attractiveness and clarity, and subsequently to higher numbers of NPs working in Swiss family practices. The wider use of EMRs and reimbursement data on NPs could facilitate future research. Further studies with larger numbers are needed to determine their exact role in Swiss primary care, their collaboration and task sharing with GPs and practice assistants, and to scrutinize the quality of care provided by NPs. For example, health insurance data could be used to assess the costs and length of NP consultations once there are separate billing options for NPs.

Our results provide first indications that NPs might have a focus on and could offer care to the growing number of multimorbid, polypharmaceutical elderly in Swiss primary care.

## Supplementary information

**Additional file 1.** Definitions of chronic conditions.

## Data Availability

The dataset is not publicly available due to the sensitivity of the data but it is available from the corresponding author on reasonable request and with permission of the participating practice.

## Abbreviations

NP: Nurse Practitioner
GP: General Practitioner
EMR: Electronic Medical Record
ICPC: International Classification for Primary Care
FIRE: Family medicine ICPC Research using EMR
