# Supplementary material for: Differences in patient population and service provision between nurse practitioner and general practitioner consultations in Swiss primary care: a case study
Source: BMC Fam Pract. 2020 Aug 13;21:164. doi: 10.1186/s12875-020-01240-8 (PMC7425147; doi:10.1186/s12875-020-01240-8)
Supplement: Supplementary file 1 — Additional file 1. Definitions of chronic conditions. [file 12875_2020_1240_MOESM1_ESM.pdf]

Definitions of chronic conditions

<table>
<tr><td>hypertension</td><td></td><td>1/0</td><td>IF atc = '^C02|^C03A|^C03EA01|^C0[78]|^C09[AB]' AND ≥2 prescriptions in ≥6 months) OR (≥2 times blood pressure systolic >140
AND Blood pressure diastolic >90)</td></tr>
<tr><td>diabetes</td><td></td><td>1/0</td><td>IF atc = '^A10'
AND ≥2 prescriptions in ≥6 months)
OR HbA1c >6.5%</td></tr>
<tr><td>cardiac_disease</td><td></td><td>1/0</td><td>IF atc = '^C01|^C03C|^C03EB01'
AND ≥2 prescriptions in ≥6 months)</td></tr>
<tr><td>epilepsy</td><td></td><td>1/0</td><td>IF atc = ''^N03A
AND NOT '^N03AE01|^N03AX16'
AND ≥2 prescriptions in ≥6 months)</td></tr>
<tr><td>hiv</td><td></td><td>1/0</td><td>IF atc = '^J05AB06|^J05AD01|^J05A[EF]'
AND ≥2 prescriptions in ≥6 months)</td></tr>
<tr><td>tuberkulosis</td><td></td><td>1/0</td><td>IF atc = '^J04A
AND ≥2 prescriptions in ≥6 months)</td></tr>
<tr><td>rheuma</td><td></td><td>1/0</td><td>IF atc = '^M01CB|^M01CC01|^P01BA02|^L01BA01|^A07EC01'
AND ≥2 prescriptions in ≥6 months)</td></tr>
<tr><td>hyperlipidemia</td><td></td><td>1/0</td><td>IF atc = '^C10' AND ≥2 prescriptions in ≥6 months)
OR Triglyzeride >1.7 mmol/l
OR Gesamt-Cholesterin >4.9 mmol/l
OR LDL-Cholesterin > 3 mmol/l
OR (sex = 'female' AND HDL-Cholesterin ≤1.2 mmol/l)
OR (sex = 'male' AND HDL-Cholesterin ≤1 mmol/l)</td></tr>
<tr><td>malignom</td><td></td><td>1/0</td><td>IF atc = ('^L01'
AND NOT '^L01BA01')
OR '^L03AA0[23]|^L03AA10|^A04AA' AND ≥2 prescriptions in ≥6 months)</td></tr>
<tr><td>parkinson</td><td></td><td>1/0</td><td>IF atc = '^N04'
AND ≥2 prescriptions in ≥6 months)</td></tr>
<tr><td>glaukoma</td><td></td><td>1/0</td><td>IF atc = '^S01E'
AND ≥2 prescriptions in ≥6 months)</td></tr>
<tr><td>dyspepsia</td><td></td><td>1/0</td><td>IF atc = '^A02[AB]'
AND ≥2 prescriptions in ≥6 months)</td></tr>
<tr><td>asthma_copd</td><td></td><td>1/0</td><td>IF atc = '^R03'
AND ≥2 prescriptions in ≥6 months)</td></tr>
<tr><td>thyroid_disease</td><td></td><td>1/0</td><td>IF atc = '^H03[AB]'
AND ≥2 prescriptions in ≥6 months)</td></tr>
<tr><td>gout</td><td></td><td>1/0</td><td>IF atc = '^M04A'
AND ≥2 prescriptions in ≥6 months)</td></tr>
<tr><td>colitis_ulcerosa</td><td></td><td>1/0</td><td>IF atc = '^A07EC'
AND NOT '^A07EC01'
AND ≥2 prescriptions in ≥6 months)</td></tr>
<tr><td>psychiatric_d</td><td></td><td>1/0</td><td>IF atc = '^N06A[ABEFGX]'| '^N05A'| '^N05B'
AND ≥2 prescriptions in ≥6 months)</td></tr>
<tr><td>adipositas</td><td></td><td>1/0</td><td>IF BMI ≥30</td></tr>
<tr><td>renal_insuff</td><td></td><td>1/0</td><td>IF eGFR < 60 (based on dkepi-calculations)</td></tr>
<tr><td>renal_insuff_end_st</td><td></td><td>1/0</td><td>IF atc = '^B03XA01|^V03AE01'
AND ≥2 prescriptions in ≥6 months)</td></tr>
</table>
